# RAF dimers control vascular permeability and cytoskeletal rearrangements at endothelial cell‐cell junctions

**DOI:** 10.1111/febs.14802

**Published:** 2019-03-18

**Authors:** Coralie Dorard, Botond Cseh, Karin Ehrenreiter, Reiner Wimmer, Andrea Varga, Tatjana Hirschmugl, Barbara Maier, Karina Kramer, Sabine Fürlinger, Eszter Doma, Manuela Baccarini

**Affiliations:** ^1^ Max F. Perutz Laboratories University of Vienna Austria; ^2^ Present address: Winnovation Consulting GmbH Vienna Austria; ^3^ Present address: Institute of Molecular Biotechnology of the Austrian Academy of Sciences Vienna Austria; ^4^ Present address: Department of Biophysics and Radiation Biology Semmelweis University Tűzoltó u. 37‐47 Budapest H‐1094 Hungary; ^5^ Present address: Ludwig Boltzmann Institute for Lung Vascular Research Graz Austria; ^6^ Present address: Mount Sinai School of Medicine New York NY USA; ^7^ Present address: Department for Health Sciences, Medicine and Research Center for Regenerative Medicine Danube University Krems Krems Austria; ^8^ Present address: Boehringer Ingelheim Pharma GmbH & Co. KG Biberach a.d. Riss Germany; ^9^ Present address: Institute of Pharmacology and Toxicology University of Veterinary Medicine Vienna Austria

**Keywords:** cell‐cell adhesions, cytoskeletal rearrangements, RAF kinases, vascular permeability

## Abstract

The endothelium functions as a semipermeable barrier regulating fluid homeostasis, nutrient, and gas supply to the tissue. Endothelial permeability is increased in several pathological conditions including inflammation and tumors; despite its clinical relevance, however, there are no specific therapies preventing vascular leakage. Here, we show that endothelial cell‐restricted ablation of BRAF, a kinase frequently activated in cancer, prevents vascular leaking as well metastatic spread. BRAF regulates endothelial permeability by promoting the cytoskeletal rearrangements necessary for the remodeling of VE‐Cadherin‐containing endothelial cell–cell junctions and the formation of intercellular gaps. BRAF kinase activity and the ability to form complexes with RAS/RAP1 and dimers with its paralog RAF1 are required for proper permeability control, achieved mechanistically by modulating the interaction between RAF1 and the RHO effector ROKα. Thus, RAF dimerization impinges on RHO pathways to regulate cytoskeletal rearrangements, junctional plasticity, and endothelial permeability. The data advocate the development of RAF dimerization inhibitors, which would combine tumor cell autonomous effect with stabilization of the vasculature and antimetastatic spread.

**Abbreviations**
AJ: adherens junction
CAB: circumferential actin bundles
EC: endothelial cells
eNOS: endothelial nitric oxide synthase
EPAC: Exchange Protein directly Activated by cAMP
eV: empty vector
FITC: Fluorescein IsoThioCyanate
GAP: GTPase‐activating protein
GEF: guanine nucleotide exchange factor
LIMK: LIM kinase
LLC‐1: Lewis lung carcinoma 1
pMECs: primary microvessel‐derived mouse endothelial cells
ROKα: RHO‐dependent kinase α
RSF: radial stress fibers
RT: room temperature
TER: transendothelial electrical resistance
VE‐Cadherin: vascular endothelial cadherin
VEGF: vascular endothelial growth factor
WT: wild‐type

## Introduction

A functioning vascular barrier is vital for many physiological processes, such as tissue‐fluid homeostasis, vascular tone, or angiogenesis 1. Endothelial cell–cell junctions are the gatekeepers of the vascular barrier, and their tight regulation is crucial for vascular function in both physiological and pathological conditions 2. Permeability‐inducing factors secreted during inflammation or tumorigenesis not only cause the efflux of protein‐rich fluid (edema) characteristic of inflammation but also the extravasation of leukocytes tasked with combating an infection 3 or of tumor cells on their way to form distant metastasis 4. These processes take place at the level of the microvasculature, where the permeability‐inducing factors locally weaken the junctions between endothelial cells by coordinated regulation of cell–cell adhesion molecules, such as VE‐Cadherin, and cytoskeletal rearrangement 5 through pathways including Src, RHO‐GTPase, or calcium signaling 1. As an example, vascular endothelial growth factor (VEGF), which plays a central role in both tumor angiogenesis and vessel permeability 6, 7, induces endothelial permeability through PLC‐dependent calcium release 8, by Src kinase‐dependent phosphorylation and internalization of VE‐Cadherin 2, 9 and by AKT/eNOS/p190RHO‐GAP (GTPase Activating Protein)‐dependent RHOA GTPase activation 10. RHO signaling also plays a key role in the induction of vascular permeability by histamine, a crucial event in allergic reactions 11 and by thrombin, which causes prolonged hyperpermeability during inflammation 12, 13. Activation of the RHO pathway by these stimuli affects F‐actin quantity and actomyosin contractility, leading to the formation of radial stress fibers (RSF) associated with junctional plasticity and intercellular gap formation. In contrast, circumferential actin bundles (CABs) strengthen cellular junctions 5, 14, 15 and must dissolve to allow their remodeling. RAP1, activated via the cAMP‐inducible GEF (Guanine nucleotide Exchange Factor) EPAC (Exchange Protein directly Activated by cAMP), prevents CAB disruption; permeability‐inducing agents such as thrombin reduce cAMP levels 16, promoting CAB weakening. Thus, induction of permeability requires fine‐tuning of RAP1 and RHO pathways, both of which must be dimmed at the junctions to allow gap formation. Simultaneously, RHO activity must increase along the RSF, at least partially through RHO GEF relocalization.

Downstream of growth factors, the RAS/RAF/MEK/ERK pathway regulates cell proliferation, migration, and survival 17. While homo‐ and heterodimerization of RAF proteins is crucial for the activation of the MEK/ERK module, RAF1 is capable of modulating parallel signaling pathways by binding and inhibiting other serine/threonine kinases, including ASK1 and ROKα. RAF1 promotes endothelial cell (EC) survival, mainly through ASK1 18, 19, 20 and regulates adherens junction (AJ) dynamics, through RAP1‐dependent binding to ROKα 21. However, the role of RAFs in endothelial permeability has not been investigated.

## Results

### Endothelial BRAF controls transendothelial resistance and paracellular permeability

**Figure 1:**
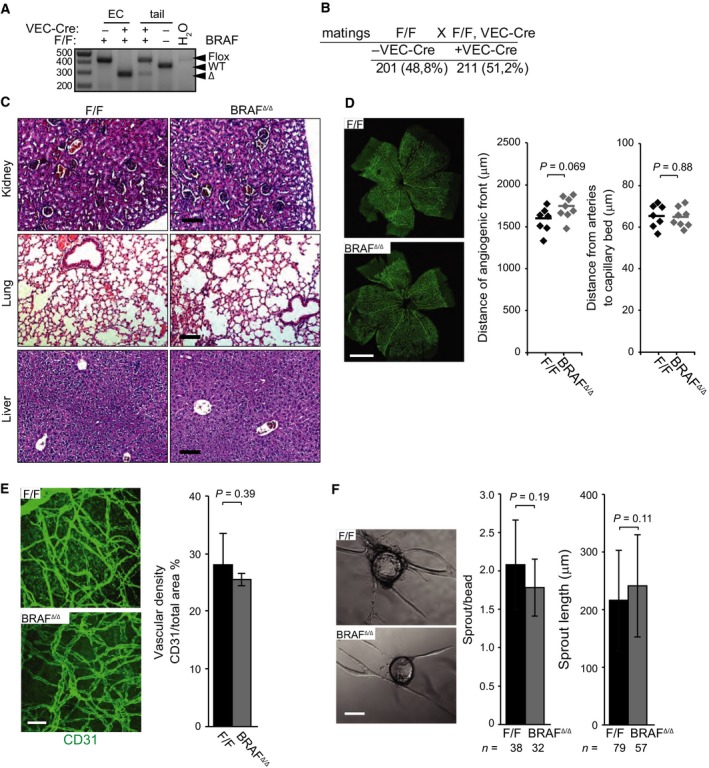
BRAF ablation does not impact embryonic development, tissue architecture, vessel maturation, and sprouting in 3D fibrin gels. (A) Efficient conversion of the flox to the Δ allele in VEC‐Cre pMECs. (B) BRAF ^Δ/Δ^ mice are viable and fertile. The number of mice recovered from F/F X F/F, VEC‐Cre intercrosses are shown. (C) BRAF ablation does not cause gross anomalies in the architecture of kidneys, lung, and liver. Tissue architecture was assessed from organs isolated from 8‐week‐old mice and stained with H&E to examine morphology. Scale bars represent 100 μm. (D) Slight increase in the progression of the angiogenic front in postnatal retinal development. The superficial vascular plexus in F/F and BRAF ^Δ/Δ^ mice is shown by tile‐scanning, composite confocal pictures of individual fields taken with a 10 × objective. Whole mounts were stained with CD31 antibody to visualize endothelial cells. Scale bar represents 1 mm. The graphs show the distance of the angiogenic front from the central optical nerve head (left) and the distance between arteries and the capillary bed (right) in (*n* = 7) F/F and (*n* = 8) BRAF ^Δ/Δ^ retinas. The *P* value was calculated according to Student's *t*‐test. (E) BRAF ablation does not influence the vascularization of subcutaneous Matrigel plugs containing FGF‐2 and VEGF (1 μg each). Whole‐mount plugs isolated from F/F (*n* = 5) and BRAF ^Δ/Δ^ (*n* = 4) mice were stained with CD31 antibody. CD31‐positive areas were quantified and are plotted in the graph. (F) BRAF ablation does not impact *in vitro* sprouting in 3D fibrin gels. pMECs were allowed to adhere to microcarriers and embedded in fibrin gels containing FGF‐2 and VEGF (200 ng·mL^−1^ each). Each pMEC sample consists of a pool of three animals. The number of sprouts/beads and the length of sprouts were microscopically assessed after 3 days in culture. The bar graphs represent means ± SD of biological replicas (E) or technical replicates (F; *n* equals the number of microcarriers and sprouts evaluated). Scale bars represent 50 μm (E) or 100 μm (F). The *P* values were calculated according to Student's *t*‐test.

We ablated *Braf* in endothelial cells by combining the *VE‐Cadherin‐Cre* (*VEC‐Cre*) transgene 22 with a homozygous *Braf* ^F/F^ allele 23. Complete conversion of *Braf* ^flox^ to *Braf* ^Δ^ was confirmed by PCR (Fig. 1A). *Braf* ^Δ/Δ^ mice (deleted in ECs) were born at Mendelian ratios (Fig. 1B), were fertile, and had a normal life span. We did not detect any anomalies in tissue architecture of *Braf* ^Δ/Δ^ kidneys, lungs, and livers (Fig. 1C). Retinal angiogenesis proceeded slightly faster in the *Braf* ^Δ/Δ^ retinas than in controls; moreover, the distance between arteries and the capillary bed was comparable in *Braf* ^Δ/Δ^ and control retinas (Fig. 1D). Thus, *Braf* ablation did not cause developmental defects or affect endothelial homeostasis. Adult angiogenesis, assessed as the ability to vascularize VEGF‐ and FGF‐containing Matrigel plugs, was similarly unaffected (Fig. 1E); and Braf^Δ/Δ^ and F/F cells performed equally well in a sprouting angiogenesis assay in 3D cultures (Fig. 1F).

**Figure 2:**
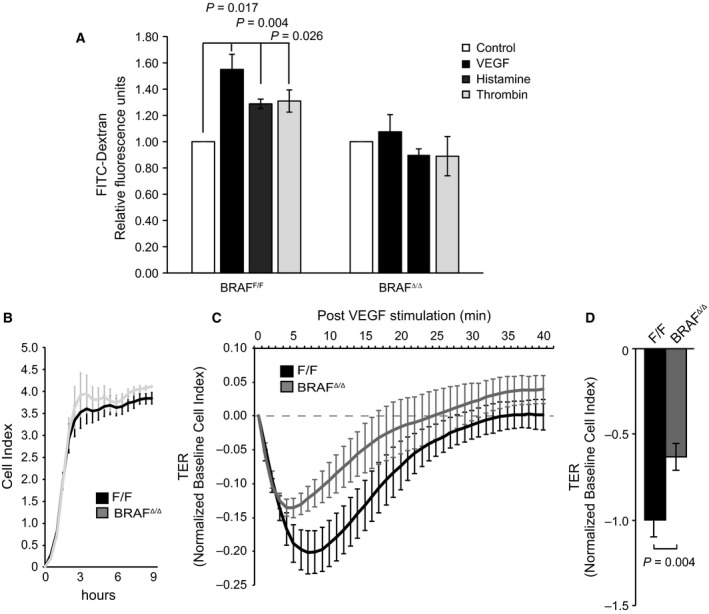
Endothelial BRAF ablation reduces paracellular permeability. (A) Decreased response of BRAF ^Δ/Δ^ pMEC monolayers to permeability‐inducing factors. Paracellular permeability was measured as the leakage of high molecular weight FITC‐Dextran across pMEC monolayers stimulated with VEGF (200 ng·mL^−1^), histamine (100 μm), or thrombin (10 U·mL^−1^). Values are normalized to PBS controls (shown as 1) and are means ± SEM of four independent experiments. (B) BRAF ablation slightly increases transendothelial resistance (TER) of pMECs monolayers, as measured by the Roche xCELLigence system. F/F and BRAF ^Δ/Δ^ endothelial monolayers’ cell index numbers were measured and compared for 9 h after plating. Values are means ± SD of 3 technical replicates. (C, D) BRAF ablation decreases the TER drop stimulated by VEGF. The data in C represent a typical plot obtained by stimulating pMEC with VEGF (200 ng·mL^−1^) or PBS 9 h after plating. Cell indexes of PBS‐treated cultures were set to 0 (dotted line; baseline values) and changes in transendothelial electrical resistance (TER) were monitored for 40 min, at which time both genotypes had returned to, or exceeded, baseline values. The data in D show a comparison of the maximum drop in TER (normalized to PBS controls) induced by VEGF in F/F and BRAF‐deficient pMECs and are means ± SD of three technical replicates. F/F values were set to −1 to allow comparison among experiments. *P* values were calculated according to Student's *t*‐test.

We next determined how BRAF ablation affected the functional properties of 2D monolayers of primary microvessel‐derived mouse endothelial cells (pMECs). VEGF‐, thrombin‐, and histamine‐induced paracellular permeability, measured by FITC‐dextran leakage 24, was significantly reduced in BRAF‐deficient pMEC monolayers (Fig. 2A).

To monitor the transient disruption of the endothelial barrier by VEGF in real time, we recorded the dynamic changes in electrical impedance (transendothelial resistance, TER) of pMEC monolayers. Figure 2B,C shows typical traces. BRAF‐deficient pMEC monolayers monitored for 9 h after plating showed a slightly higher baseline cell index than F/F cultures (Fig. 2B). VEGF treatment caused a transient drop in TER which was less pronounced and more transient in BRAF‐deficient monolayers (Fig. 2C,D), indicating increased endothelial barrier function in good agreement with the results of the paracellular permeability assay (Fig. 2A).

### BRAF ablation impacts signaling to the cytoskeleton

**Figure 3:**
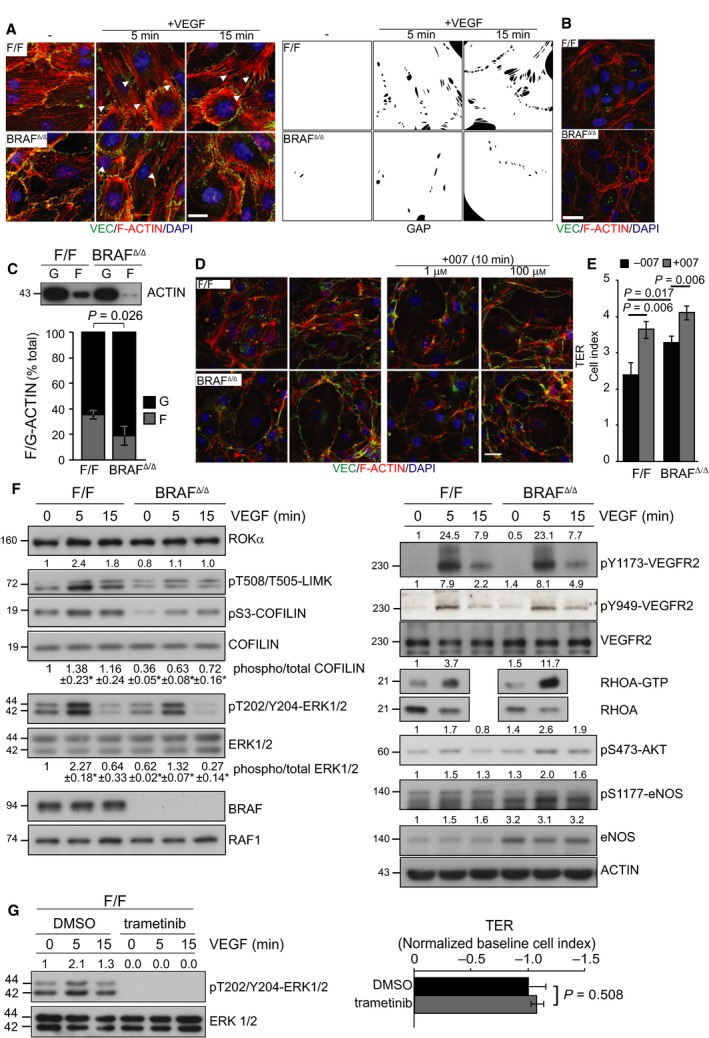
BRAF ablation reduces intercellular gap formation and VEGF‐induced signaling to the cytoskeleton independently of ERK. (A) Intercellular gap and RSF formation induced by VEGF (50 ng·mL^−1^) are decreased in BRAF‐deficient pMEC monolayers. Arrows indicate intercellular gaps. The staining shows VE‐Cadherin (green), F‐actin (phalloidin, red) and cell nuclei (DAPI, blue). Scale bar represents 20 μm. The bottom panel shows a silhouette representation showing the gaps in black. B, Reduced RSF and increased CABs in quiescent BRAF ^Δ/Δ^ pMEC monolayers. F‐actin (phalloidin, red), VE‐Cadherin (green), and nuclei (DAPI, blue) staining are shown. Scale bar = 20 μm. The magnetic beads used for purifying the pMECs autofluoresce in green. C, Reduced F/G‐actin ratio in BRAF ^Δ/Δ^ pMECs. Filamentous and globular actin from F/F and BRAF ^Δ/Δ^ pMECs were separated by ultracentrifugation and their percentage was determined by immunoblotting. The bars represent the mean ± SD of immunoblots from three independent experiments analyzed using the imagej software. (D, E) the EPAC/RAP1 activator 007 changes the morphology and increases transendothelial resistance (TER; measured as in Fig. 2C) of F/F pMEC monolayers, rendering them more similar to BRAF‐deficient pMECs. D, F‐actin (phalloidin, red), VE‐Cadherin (green) and nuclei (DAPI, blue) staining are shown. Scale bar = 20 μm. The magnetic beads used for purifying the pMECs autofluoresce in green. (E) The plot shows F/F and BRAF ^Δ/Δ^ endothelial monolayers’ cell index numbers measured before (−007; minimum values) and after 007 treatment (+007, maximum values). Values are means ± SD of three technical replicates. (F) VEGF signaling is perturbed by BRAF ablation. Lysates from F/F and BRAF ^Δ/Δ^ pMECs stimulated with 200 ng·mL^−1^ VEGF were analyzed by immunoblotting using the indicated antibodies. RHOA activation was determined as the proportion of GTP‐loaded protein. (G) Trametinib (10 nm, 1 h prior to VEGF addition) efficiently inhibits MEK/ERK (left panel) but does not phenocopy the decreased VEGF‐stimulated TER of BRAF‐deficient pMECs (right panel). Values represent the maximum VEGF‐induced drop in TER (normalized to PBS controls) and are means ± SD of three technical replicates. DMSO‐treated pMECs were set to −1 to allow comparison. The numbers above the blots show the quantification of the specific experiments shown, while the values underneath the COFILIN and the ERK panels show quantifications of pCOFILIN and pERK levels obtained in three independent experiments, normalized to the phospho/total levels of unstimulated F/F pMECs, set as 1 (*P < 0.045). *P* values were calculated according to Student's *t*‐test.

To gain more insight into the mechanism by which BRAF regulates paracellular permeability, we monitored morphological changes in monolayers of pMEC continuously growing, starved, or exposed to VEGF. VEGF induced RSF formation, elongation of VE‐Cadherin‐containing AJs (indicative of radial tension), and intercellular gap development in F/F pMECs, but were severely impaired in BRAF‐deficient pMECs (Fig. 3A). These qualitative results are consistent with, and complement, the quantitative measurement of barrier function (Fig. 2A,C,D). Notably, the reduction in RSF and prominent CAB were stimulus‐independent and could also be observed in unstimulated or continuously growing BRAF‐deficient pMECs (compare Fig. 3A,B), where they also correlated with reduced F‐actin content (Fig. 3C). The morphology of BRAF‐deficient pMECs was similar to that of F/F cells treated with the EPAC activator 007 (Fig. 3D), which decreases permeability of endothelial monolayers through a RAP1‐dependent tightening of VE‐Cadherin‐containing AJ 14, 15, 25. Treatment with 007 significantly increased TER in both F/F and BRAF‐deficient MECs; however, there was no significant difference between 007‐treated F/F pMECs and untreated BRAF‐deficient pMECs, indicating that 007 treatment and BRAF ablation have a similar impact on AJ tightening (Fig. 3E).

In good correlation with the reduction in RSF and F‐actin and with the prominent CAB observed in continuously growing, unstimulated or VEGF‐treated BRAF‐deficient pMECs, we observed a decrease in the phosphorylation of the ROKα (RHO‐dependent kinase α) effector LIMK (LIM Kinase) and of its target, the actin‐severing protein COFILIN, used as a readout for ROK signaling, under both basal and VEGF‐induced conditions (Fig. 3F, left panel). VEGF signaling upstream of ROK was unaltered or slightly increased in BRAF‐deficient pMECs compared with F/F cells (Fig. 3F, right panel), suggesting a roadblock in RHOA signaling at the level of ROKα. Reduced COFILIN phosphorylation, RSF formation, and F‐actin content have also been observed in BRAF knockout fibroblasts, where they correlated with ERK‐dependent reduction in ROKα expression 26. ROKα expression, however, was indistinguishable in BRAF‐proficient and ‐deficient pMECs (Fig. 3F), indicating that a distinct mechanism impacts ROKα signaling in the latter cell type. BRAF could also promote actomyosin formation, cell contractility 27, and endothelial permeability 28 through its effectors MEK/ERK, which activate MLCK (myosin light chain kinase) 29. VEGF‐induced ERK activation was reduced in BRAF‐deficient pMEC monolayers (Fig. 3F). However, the MEK inhibitor trametinib, which completely blunted ERK activation in F/F pMECs, had no impact on VEGF‐induced loss of TER (Fig. 3G), indicating that the reduced MEK/ERK activation in BRAF‐deficient pMEC is not the cause of decreased permeability.

### BRAF ablation increases RAF1 interaction with ROKα at VE‐Cadherin‐containing AJs

**Figure 4:**
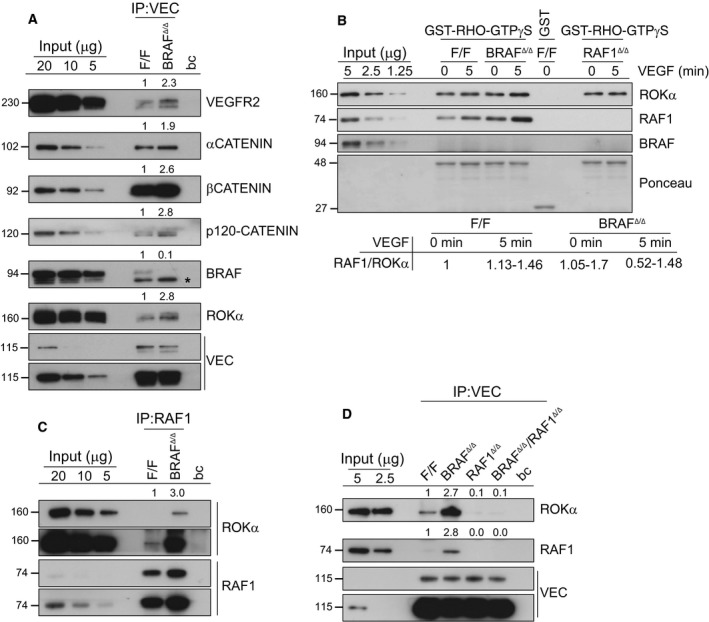
BRAF ablation increases RAF1 interaction with ROKα at VE‐Cadherin‐containing junctions. (A) BRAF ablation promotes the association of VEC with VEGFR2, Catenins, and ROKα. The asteriks (*) marks an unspecific band in the BRAF blot. (B) RAF1 is recovered with ROKα in RHOA‐GTP pull downs. The ability of ROKα to bind to active RHOA was determined by pull down with GST‐RHOA‐GTPγS from lysates of F/F, BRAF ^Δ/Δ^ and RAF1^Δ/Δ^ pMEC stimulated with 200 ng·mL^−1^ VEGF. RHOA‐binding proteins were detected by immunoblotting. The range of two experiments is shown in the table underneath the blot. (C) BRAF ablation increases the association of RAF1 with ROKα. (D) The association of ROKα with VEC depends on the presence of RAF1. VEC (A and D) or RAF1 (C) immunoprecipitates were prepared from F/F and BRAF ^Δ/Δ^ pMEC monolayers. In A, C, and D, the presence of VEC or RAF1 and coimmunoprecipitating proteins were detected by immunoblotting. The numbers above the blots show the quantification of the specific experiments shown, performed by normalizing the amount of coimmunoprecipitated proteins to the amount of immunoprecipitated antigen. The value of the F/F cells was set as 1. ‘bc’ refers to beads control (A, C, D).

The VE‐Cadherin‐containing junctions are crucial for the regulation of vessel permeability. Association of VE‐Cadherin with VEGFR2 induces its endocytosis, destabilizing the junctions; in contrast, the association with the cytoskeleton and particularly with CAB increases AJ stability 30. Consistent with the decreased sensitivity to permeabilizing agents and with the prominent CAB observed in BRAF‐deficient pMECs, VE‐Cadherin association with VEGFR2 and with the cytoskeleton (measured by binding to α, β, and p120 catenins; Fig. 4A) was increased in these cells. Low amounts of BRAF could be detected in F/F VE‐Cadherin immunoprecipitates; importantly, however, the association of VE‐Cadherin with ROKα was increased (2.8‐fold) in BRAF‐deficient pMECs (Fig. 4A; see also Fig. 4D). ROKα binding to recombinant RHOA‐GTPγS was not decreased in BRAF‐deficient lysates, indicating that this is not the activation step inhibited by BRAF ablation. The ROKα interactor RAF1, but not BRAF, could be recovered in the RHOA‐GTPγS pull downs (Fig. 4B). Similar amounts of ROKα were recovered in RHOA‐GTPγS pull downs from control and RAF1‐deficient lysates; thus, RAF1 is dispensable for the binding of ROKα to active RHOA. BRAF ablation slightly increased the amount of RAF1 present in the RHOA‐GTPγS pull downs; consistently, more ROKα was present in RAF1 immunoprecipitates from BRAF‐deficient than from F/F pMECs (Fig. 4C, threefold increase). The amount of RAF1 and ROKα interacting with VE‐Cadherin was also increased to a similar extent (2.8‐fold) in BRAF‐deficient pMECs, as shown by VE‐Cadherin immunoprecipitation (Fig. 4D; see also Fig. 4A). Consistent with our previous results 21, ROKα interaction with VE‐Cadherin was RAF1‐dependent, and was abrogated in pMECs with compound BRAF and RAF1 ablation (BRAF^Δ/Δ^/RAF1^Δ/Δ^; Fig. 4D). BRAF^Δ/Δ^/RAF1^Δ/Δ^ pMECs were isolated from BRAF^Δ/Δ^/RAF1^Δ/Δ^ mice, which do not show any apparent developmental defects or advantages (data not shown). The pMECs grew normally in culture.

### RAF1 ablation rescues the permeability defects of BRAF‐deficient pMECs

**Figure 5:**
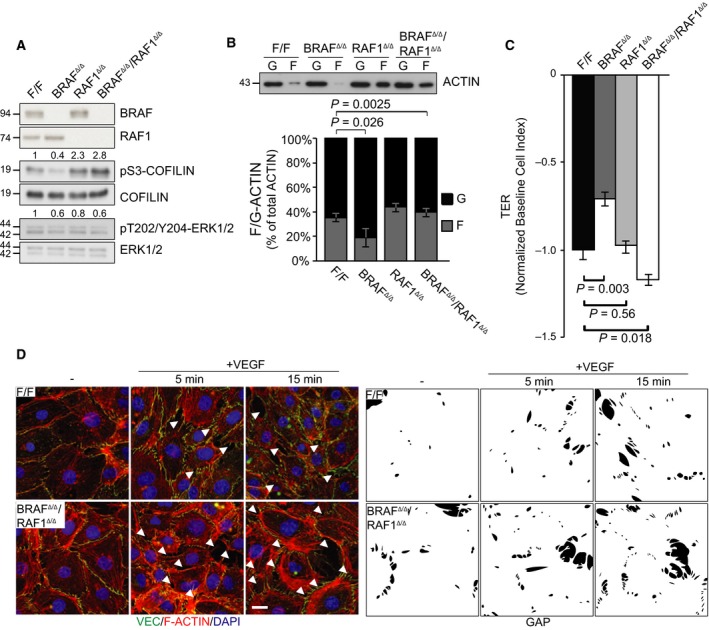
RAF1 ablation rescues the molecular and cellular phenotypes of BRAF ^Δ/Δ^ pMECs. (A) Increased COFILIN and reduced ERK phosphorylation in BRAF ^Δ/Δ^/RAF1^Δ/Δ^ pMEC monolayers. Lysates were analyzed by immunoblotting using the indicated antibodies. The numbers above the blot show the quantification of the specific experiment shown. (B) RAF1 ablation increases the ratio of F/G‐actin in pMECs. Filamentous and globular actin from F/F (*n* = 3), BRAF ^Δ/Δ^ (*n* = 3), RAF1^Δ/Δ^ (*n* = 2), and BRAF ^Δ/Δ^/RAF1^Δ/Δ^ (*n* = 3) pMECs were separated by ultracentrifugation and analyzed by immunoblotting. (C) RAF1 ablation rescues the permeability defect of BRAF‐deficient pMEC monolayers treated with 200 ng·mL^−1^ VEGF. TER was measured as described in the legend to Fig. 2. Values represent the maximum VEGF‐induced drop in TER (normalized to PBS controls) and are means ± SD of three technical replicates. F/F values were set to −1 to allow comparison among experiments. *P* values were calculated according to Student's *t*‐test. (D) RAF1 ablation normalizes intercellular gap formation induced by 50 ng·mL^−1^ VEGF in BRAF ^Δ/Δ^ pMEC monolayers. Arrows indicate intercellular gaps. The staining shows VE‐cadherin (green), F‐actin (phalloidin, red), and cell nuclei (DAPI, blue). Scale bar represents 20 μm. The bottom panel shows a silhouette representation showing the gaps in black.

We next investigated whether increased RAF1/ROKα interaction and recruitment to VE‐Cadherin observed in BRAF‐deficient pMECs was causally linked to the decrease in COFILIN phosphorylation, filamentous actin, RSF, and TER. In BRAF^Δ/Δ^/RAF1^Δ/Δ^ pMECs, ERK phosphorylation was decreased to a level comparable to that of BRAF^Δ/Δ^ pMECs (Fig. 5A). The residual ERK phosphorylation in BRAF^Δ/Δ^/RAF1^Δ/Δ^ pMECs does not correlate with ARAF upregulation (data not shown). A similar phenotype has been observed in primary keratinocytes 31, 32, 33, and may be due to the activity of alternative MEK kinases, such as TPL2 or MOS, due to reduced DUSP expression, or due to the attenuation of ERK‐dependent negative feedback mechanisms.

As previously described for RAF1^Δ/Δ^ pMECs 21, COFILIN phosphorylation was higher in BRAF^Δ/Δ^/RAF1^Δ/Δ^ pMECs than in control cells. Consistently, F‐actin content was increased in RAF1^Δ/Δ^ and BRAF^Δ/Δ^/RAF1^Δ/Δ^ pMECs (Fig. 5B). Rescue of the cytoskeletal phenotype was accompanied by restored VEGF‐induced permeability, as quantitated by TER (Fig. 5C), and intercellular gap formation (Fig. 5D). Thus, BRAF/RAF1 ablation rescues the permeability defects of BRAF^Δ/Δ^ pMECs, and phenocopies those of RAF1^Δ/Δ^ pMECs 21.

### RAF dimerization regulates VEGF‐induced permeability and cytoskeletal rearrangements

**Figure 6:**
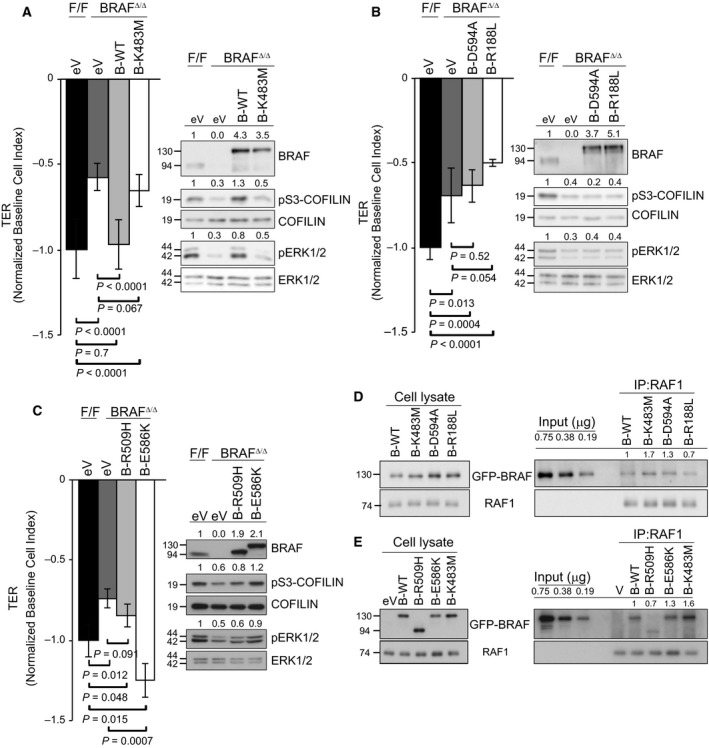
BRAF kinase activity, RAS binding, and RAF dimerization are necessary for the regulation of VEGF‐induced permeability. BRAF ^Δ/Δ^ pMECs were reconstituted with empty vector (eV) or with the following GFP‐tagged BRAF constructs; wild‐type (B‐WT) in A; kinase dead (B‐K483M in A and B‐D594A in B); RAS‐binding deficient (B‐R188L in B); RAF dimerization mutants (B‐R509H, reduces RAF dimerization; and B‐E586K, promotes RAF dimerization) in C. VEGF‐induced permeability was monitored by TER (left panels). Values represent the maximum VEGF‐induced drop in TER (normalized to PBS controls) and are means ± SD of ≥ 3 technical replicates. F/F values were set to −1 to allow comparison among experiments. COFILIN and ERK phosphorylation (right panels) in the total lysates of the transfected cells were determined by immunoblotting. (D, E) RAF1 immunoprecipitates were prepared from COS7 cells reconstituted with WT‐BRAF, BRAF‐K483M, BRAF‐D594A, and BRAF‐R188L (D) or WT‐BRAF, BRAF‐R509H, BRAF‐E586K, and BRAF‐K483M (E). RAF1 and coimmunoprecipitating BRAF were detected by immunoblotting. The panels on the left show the expression levels of the different constructs. The numbers above the blots show the quantification of the specific experiments shown.

To gain insight on the mechanism by which BRAF impacts the binding of RAF1 to ROKα, we transfected pMECs either with empty vector (eV) or with constructs encoding wild‐type (WT) or kinase‐dead (K483M) BRAF proteins 34. Wild‐type BRAF, but not the K483M mutant, efficiently rescued permeability and increased both COFILIN and ERK phosphorylation (Fig. 6A). These results were confirmed using a second kinase‐dead mutant (D594A; Fig. 6B) 34. Additionally, a BRAF mutant which cannot bind to RAS or RAP1 (R188L) 35 failed to rescue both the biological and the biochemical phenotypes of BRAF‐deficient pMECs (Fig. 6B). Thus, both RAS/RAP1 binding and BRAF kinase activity are required for the control of pMEC permeability by BRAF. We analyzed the significance of RAF dimerization in the control of pMEC permeability by BRAF using mutants with either reduced (R509H) or increased (E586K) affinity for RAF1 (Fig. 6C) 36. R509H BRAF failed to rescue the TER phenotype and led to a marginal increase in pCOFILIN and pERK. Conversely, E586K significantly increased VEGF‐induced permeability as well as COFILIN and ERK phosphorylation (Fig. 6C). As confirmed in cotransfected COS7 cells, the ability of BRAF mutants to dimerize with RAF1 correlated with their ability to rescue the endothelial cell phenotype (Fig. 6D,E). Collectively, the data indicate that the role of BRAF in permeability is kinase dependent and that it requires RAS/RAP1 binding and dimerization with RAF1.

### Endothelial BRAF controls vessel permeability *in vivo*

**Figure 7:**
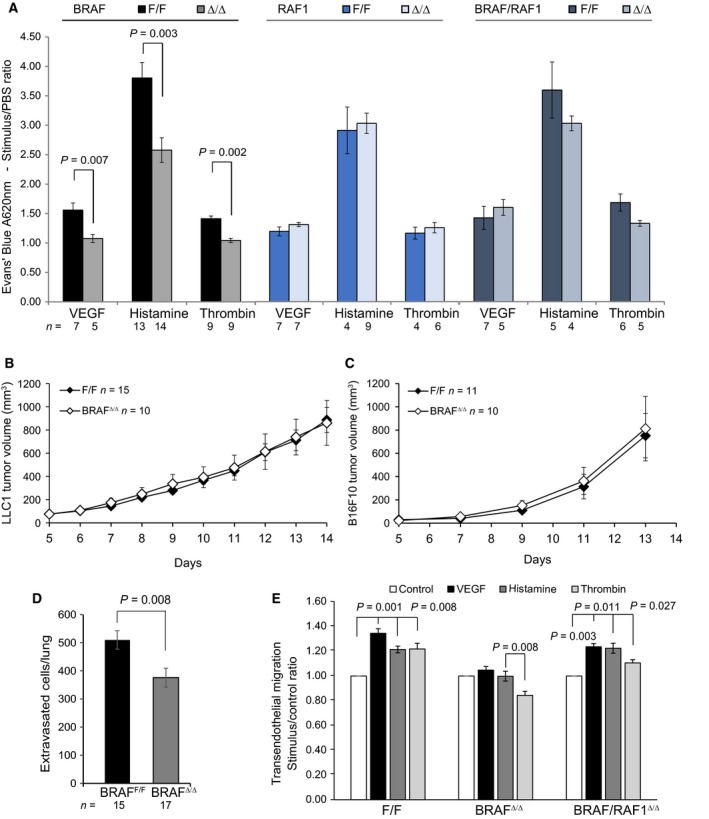
Endothelial BRAF ablation reduces vascular permeability and cell extravastation *in vivo* in a RAF1‐dependent manner. (A) Reduced dermal vascular permeability in response to permeability stimuli in BRAF ^Δ/Δ^, RAF1^Δ/Δ^, and BRAF/RAF1^Δ/Δ^ animals. Quantification of dermal vascular permeability after intradermal injection of VEGF (400 ng), histamine (1 μg), thrombin (10 U), or PBS into F/F and BRAF ^Δ/Δ^ mice. Evans Blue dye leakage is plotted as stimulus/PBS ratio (mean ± SEM). (B, C) BRAF ^Δ/Δ^ mice support the growth of Lewis lung carcinoma (LLC‐1, B) and B16F10 melanoma (C) allografts. Tumor volumes were assessed at the indicated days after subcutaneous implantation of 10^6^ cells into F/F or BRAF ^Δ/Δ^ animals. Tumor‐bearing mice were sacrificed 14 (LLC1) or 13 (B16F10) days after injection of tumor cells. (D) Reduced extravasation of CMRA‐labeled B16F10 melanoma cells following tail vein injections in BRAF ^Δ/Δ^ mice. The number of extravasated B16F10 cells in the lungs of F/F and BRAF ^Δ/Δ^ animals was quantified 48 h after injection. The data represent average values ± SEM of the indicated biological replicates. (E) Reduced transendothelial migration of B16F10 melanoma cells through BRAF ^Δ/Δ^ pMEC, but not BRAF/RAF1^Δ/Δ^ monolayers. CMRA‐labeled B16F10 melanoma cells were allowed to migrate through confluent pMEC monolayers on fibronectin‐coated transwell membranes. Transmigrated cells were counted after a 6‐h incubation with the indicated stimuli. The plots represent the mean (± SEM) of four independent experiments, each performed in triplicates. Values are normalized to PBS controls (shown as 1). A, D, and E *P* values were calculated according to Student's *t*‐test.

To determine whether BRAF was required for the control of endothelial permeability *in vivo*, we next injected VEGF, histamine, or thrombin, all of which act through the RHO/ROK signaling pathway 10, 11, 12, 13, intradermally in *Braf* ^*Δ/Δ*^, and control littermates. BRAF‐deficient vessels were more resistant to all three permeability‐inducing stimuli; however, intradermal injection of VEGF, histamine, or thrombin induced similar levels of permeability in RAF1^Δ/Δ^, BRAF^Δ/Δ^/RAF1^Δ/Δ^, and control mice (Fig. 7A). To assess whether the permeability phenotype impacts tumor growth, we used two different allograft models that depend on tumor vascularization, namely Lewis lung carcinoma (LLC‐1) and B16F10 melanoma grafts, which depend on VEGF for growth 37, 38. *Braf* ^*Δ/Δ*^ and F/F littermate supported the growth of LLC‐1 and B16F10 grafts at indistinguishable levels (Fig. 7B,C). However, colonization of the lung by B16F10 melanoma cells injected in the tail vein, a widely used model for tumor cell extravasation in the lung vasculature 39, was less efficient in *Braf* ^*Δ/Δ*^ than in control littermates (Fig. 7D). Consistently, VEGF, histamine, and thrombin also promoted the migration of B16F10 melanoma cells through a monolayer of F/F, but not BRAF knockout endothelial cells, and this phenotype was rescued in BRAF/RAF1 knockout monolayers (Fig. 7E).

**Figure 8:**
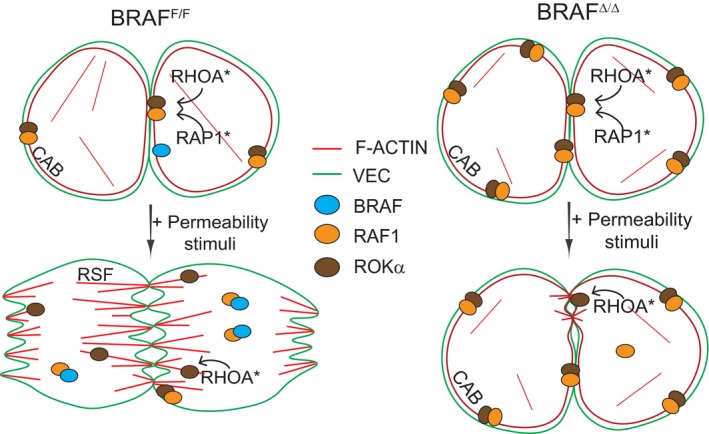
A BRAF/RAF – RAF1/ROKα rheostat regulates paracellular permeability in endothelial monolayers. Working model (see text for detail). In endothelial cells, RAP1‐dependent localization of the RAF1/ROKα complex to VE‐Cadherin‐containing AJs and localized RHOA signaling favor CAB formation and junctional stability. BRAF/RAF1 dimerization antagonizes this, decreasing RHOA/ROKα signaling at the AJs. This allows the disruption of CAB and promotes the formation of RSFs, AJ remodeling, and the formation of intercellular gaps when permeabilization is induced (RHOA* active RHOA; RAP1* active RAP1). In the absence of BRAF, more RAF1/ROKα complexes colocalize with VEC, reinforcing CAB formation and cell–cell junctions, and resulting in decreased vessel permeability.

Taken together, the results show that endothelial BRAF ablation reduces the paracellular permeability of endothelial monolayers in culture and vessel permeability *in vivo* irrespectively of the inducer, and that these phenotypes depend on the presence of RAF1 and on the formation of BRAF/RAF1 dimers (Fig. 8).

## Discussion

Vascular permeability defects are common to many pathological conditions. Weakening of the endothelial barrier causes vascular leakage and edema in cardiovascular and inflammatory diseases. In cancer, the leaky tumor‐associated vasculature facilitates metastatic spreading and hampers drug delivery. In both instances, normalization of the vasculature would be desirable; the search for therapeutic approaches based on the molecular understanding of the endothelial barrier function is ongoing.

### A BRAF/RAF–RAF1/ROKα rheostat regulates paracellular permeability in endothelial monolayers

The regulation of AJ and cytoskeletal remodeling by RHO GTPases play a crucial role in endothelial permeability. Specifically, RAP1 and RHO have opposite functions, the former stabilizing CAB and AJs, the latter driving RSF formation, contractility, and AJ remodeling 2, 30. Permeability‐perturbing agents cause activation and relocalization of RAP1 and RHO through their activators (GEFs) or inhibitors (GAPs). While this part of the signaling pathways leading to paracellular permeability is rather well studied 40, what happens downstream is less clear.

Our data now show that BRAF controls endothelial permeability by reducing both the binding of RAF1 to ROKα and the recruitment of this complex to VE‐Cadherin‐containing AJs. All players are found in complex with VE‐Cadherin (Fig. 4). In BRAF‐deficient pMECs, increased ROKα signaling at the AJs favors the formation of CAB over RSF and reduces overall F‐actin content. These morphological and biochemical phenotypes are evident in unstimulated pMECs. In contrast, the physiological phenotype is revealed both *in vivo* and *in vitro* by stimulation with permeability‐inducing agents. In BRAF‐deficient cells and vessels, the efficacy of these agents is reduced due to the stabilization of AJs and the increased strength of the tonic permeability barrier induced by increased RAF1/ROKα signaling.

This conclusion is backed by the fact that only BRAF proteins able to bind to RAF1 are able to rescue the permeability phenotype in pMEC monolayers; equally importantly, the phenotypes of BRAF‐deficient cells are rescued by the concomitant ablation of RAF1. By demonstrating that BRAF, RAF1, and ROKα receive and integrate signals from permeability stimuli, and that BRAF/RAF1 and RAF1/ROKα heterodimers act as a rheostat fine‐tuning endothelial barrier function, our results advance our understanding of the mechanisms modulating AJ dynamics and cytoskeletal remodeling.

### Potential mechanisms of BRAF/RAF1 and RAF1/ROKα heterodimerization

We have recently shown that the RAF1 phosphospecies able to bind ROKα is generated in the context of RAF dimers formed during ERK activation. However, in the context of the RAF dimer, BRAF promotes RAF1 autophosphorylation on a 14‐3‐3 residue which stabilizes RAF dimers, thereby favoring BRAF/RAF dimerization over RAF1‐ROKα complex formation and efficiently controlling their levels 41. How exactly BRAF/RAF1 dimerization is modulated by permeability‐promoting signals in pMECs is unclear. RAS activation, which regulates different aspects of endothelial cell biology 42, 43, 44, occurs upon stimulation with VEGF but also with thrombin 45 and, at least in HEK293T cells, with histamine 46. Alternatively, RAP1, which has been shown to regulate both RAF1/ROKα heterodimerization and their association with VE‐Cadherin at AJs 21, may also control RAF dimerization. In favor of this, RAP1 activates ERK via BRAF 47, 48, activates BRAF in cell‐free extracts 49 and binds to both RAF molecules with different affinities, determined by their divergent CRD domains 50.

In this scenario, both BRAF/RAF1 and RAF1/ROKα heterodimers would be stimulated by the activation of the same GTPase, RAP1 (Fig. 8).

### But if this is the case, how do RAF1/ROKα heterodimers form in BRAF‐deficient cells?

It is important to point out here that low levels of basal and growth factor‐induced ERK phosphorylation are still detectable in BRAF‐deficient pMEC (Fig. 4), fibroblasts, and keratinocytes 51, indicating that this function of BRAF is at least partially redundant. It is thus likely that other RAF1 dimerization partners (such as RAF1 itself, ARAF, or KSR) can both maintain ERK activation and prime RAF1 for ROKα complex formation in BRAF‐deficient cells. Over time, the interaction with these less efficient dimerization partners/activators would generate an increased number of ROKα‐binding RAF1 molecules, leading to the cytoskeletal phenotypes observed in BRAF‐deficient pMECs. In favor of this hypothesis, increased RAF1/ROKα complex formation has also been observed in BRAF‐deficient keratinocytes 31.

Whatever the precise mechanism underlying their yin‐yang behavior in pMECs, the BRAF/RAF1 –RAF1/ROKα module impacts permeability induced by agents responsible for vessel leakage not only in tumors but also in other conditions, including cardiovascular and inflammatory diseases. Our results thus suggest that inhibitors preventing RAF dimerization would be beneficial in a broad range of disorders associated with permeability defects. In the specific context of cancer, RAF dimerization inhibitors combine a beneficial cell autonomous effect on tumor proliferation, by reducing the activity of the ERK pathway, with the normalization of vascular permeability, allowing for better drug delivery.

## Methods

### Generation of BRAF^Δ/Δ^ mice

BRAF^F/F^ mice were mated to VEC‐Cre 22 (Charles River Laboratories, Sulzfeld, Germany) mice to obtain BRAF^Δ/Δ^ animals. BRAF ablation was determined by allele‐specific PCR analysis as previously described 21. Compound deletion of BRAF and RAF1 in endothelial cells was obtained by mating BRAF^F/F^/RAF1^F/F^ mice with VEC‐Cre‐expressing animals. Animal experiments were authorized by the Austrian Ministry of Science and Communications, following the approval by the national Ethical Committee for Animal Experimentation.

### Retinal angiogenesis

Whole‐mount retinas derived from 6‐day‐old animals were stained with CD31 antibody (BD Pharmingen, BD Biosciences, Franklin Lakes, NJ, USA; cat. No. 550274) to visualize the vascular plexus 21 and quantify the distance between central optical nerve head and angiogenic front and between capillaries and arteries.

### Matrigel plug assay

400‐μL high concentration Matrigel, (BD Bioscience) supplemented with 1 μg of recombinant human FGF‐2 and 1 μg VEGF (R&D Systems, Minneapolis, MN, USA) was injected subcutaneously in the flank of the mice. Matrigel plugs were isolated 10 days postinjection, fixed in 4% PFA or frozen in Tissue‐Tek^®^ O.C.T™ Compound (Sakura FineTek, Torrance, CA, USA) and analyzed by immunohistochemistry.

### Histology

Hematoxylin/eosin staining was performed on 3‐μm‐thick paraffin sections of 4% paraformaldehyde‐fixed tissue. Vascular density was determined by staining cryo‐embedded, 50‐μm‐thick tumor sections with anti‐CD31 (BD Pharmingen).

### pMEC isolation, culture, and transfection

The pMECs used throughout this study were isolated from collagenase‐digested lungs of 10‐day‐old mice, enriched by two rounds of sorting with ICAM‐2 (BD Pharmingen) coupled to dynabeads (Dynal Biotech, Invitrogen, Carlsbad, CA, USA; 1 h at 4 °C) and cultured in EC culture medium [DMEM plus nonessential amino acids, 1 mm sodium pyruvate (Gibco, Life Technologies, Gaithersburg, MD, USA), 25 mm HEPES pH 7.4 (Sigma‐Aldrich, St. Louis, MO, USA), penicillin/streptomycin, and 20% FBS (Sigma) + 100 μg·mL^−1^ Endothelial Mitogen (Merck Millipore, Billerica, MS, USA) and 20 U·mL^−1^ Heparin (Sigma)] as previously described 21. Each pMEC sample represents a pool of three animals. The protocol reproducibly yields 95–98% pure pMECs 52. pMECs were transfected with pcDNA3.1 (Invitrogen) containing GFP‐tagged BRAF constructs (BRAF WT, gift of Richard Marais, CRUK, Manchester; and mutants generated by site‐directed mutagenesis) using poly(ethylenimine) (Sigma) in accordance with the manufacturer's protocol, and used in TER measurements 16–18 h later. For growth factor stimulation, pMECs were incubated in FBS‐reduced medium (1% FBS) for 16–18 h prior to treatment with VEGF at the concentration and for the time indicated.

### Fibrin gel bead assay

*In vitro* 3D sprouting of pMECs was carried out as described previously 21. Briefly, 2500 Cytodex beads (GE Healthcare, Pittsburgh, PA, USA) were incubated with 10^6^ pMECs and plated overnight on a 10‐cm dish to remove unattached cells. Next day, 1000 cell‐covered beads were resuspended in 2 mg·mL^−1^ fibrinogen (Sigma) solution containing 0.15 U·mL^−1^ aprotinin (Sigma), 200 ng·mL^−1^ FGF‐2, and 200 ng·mL^−1^ VEGF, mixed with Thrombin (Sigma; 0.625 U·mL^−1^), allowed to clot in 24‐well plates (5–10 min) and covered with EC base medium. Sprout formation was imaged with a Zeiss Axiovert 200M equipped with an Axiocam MRm and analyzed with the zeiss axiovision software (Zeiss, Jena, Germany).

### Paracellular permeability assays

The FITC‐Dextran permeability assay was performed by adding FITC‐Dextran (± 200 ng·mL^−1^ VEGF, 100 μm histamine or 10 U·mL^−1^ thrombin) to pMECs monolayers cultured on fibronectin‐coated semipermeable inserts (0.4 μm pore size) and measuring its passage to the lower compartment after 1 h, according to the supplier's protocol (Millipore). Changes in the transendothelial electrical resistance (TER) of pMEC monolayers were measured using xCELLigence system (RTCA‐DP version; Roche Diagnostics, Mannheim, Germany), which tracks changes in electrical impedance (expressed as “cell index”, proportional to cell attachment and spreading). Permeability‐inducing agents causing the appearance of intercellular gaps result in changes in electrical impedance quantifiable in real time. pMECs were plated (1.5 × 10^5^) and allowed to grow to confluence overnight on fibronectin‐coated 96‐well E‐plates prior to the addition of PBS or permeability‐modifying agents [200 ng·mL^−1^ VEGF or 100 μm 007 (8‐pCPT‐2′‐O‐Me‐cAMP; Biolog Life Science, Bremen, Germany)] 15. For MEK inhibition, cells were pretreated with 10 nm trametinib for 1 h before the addition of VEGF. To compare the effect of permeability‐inducing stimuli on the different genotypes, the cell index recorded at the time of addition of the permeability‐inducing stimuli or their vehicles was set as 0, and the changes in cell index induced by the permeability stimuli were subtracted from those obtained by treating the cells with their vehicles. This normalization is necessary because the cell indexes of unstimulated F/F and BRAF^Δ/Δ^ (raw data) differ slightly (see Fig. 2B). Thus, the drop of TER caused by permeability‐inducing stimuli appears as a negative value. To further help comparison among experiments and different stimuli, the values representing the maximum drop in TER induced by the stimuli in wild‐type pMEC monolayers are normalized to −1 in all plots except Figs 2B and 3E, in which cell index is shown instead, and Fig. 2C, which shows the full kinetics of VEGF‐induced permeability.

### Immunofluorescence and filamentous (F):globular (G) actin ratios

Cells were permeabilized (0.2% Triton X‐100 in PBS, 15 min RT), blocked (3% FCS in PBS, 30 min RT), and washed extensively with PBS prior to the incubation with rat anti‐mouse‐VEC (BD Pharmingen) antibody (1 : 100 in 3% FCS, overnight at 4 **°**C). After thorough washing in PBS, cells were stained simultaneously with the anti‐rat‐Alexa Fluor488 and Alexa Fluor594 Phalloidin (both Invitrogen; 1 : 500 in 3% FCS, 1 h at RT), washed in PBS, counterstained with DAPI, and mounted in Prolong Gold Antifade Reagent (Life Technologies, Carlsbad, CA, USA). Images were acquired with an inverse spinning disk (Visitron, Puchheim, Germany) equipped with a sensitive EM‐CCD camera (Hamamatsu ImageEM X2, Hamamatsu, Japan) and a Plan‐Apochromat 63×/1.4 Oil DIC objective lens, and analyzed with the imagej software (NIH, National Institute of Health, Bethesda, Maryland, USA).

The F‐actin/G‐actin ratio was determined using an assay kit (Cytoskeleton, Denver, CO, USA) according to the supplier's protocol. Briefly, pMECs were lysed in 500 μL detergent‐based lysis buffer and subjected to an ultracentrifugation step which pellets F‐actin and leaves G‐actin in the supernatant. The amount of actin in supernatant and pellet was determined by immunoblotting.

### Immunoprecipitation, pull down, and immunoblotting

For immunoprecipitation, cells lysates prepared in a buffer containing 25 mm HEPES, 150 mm NaCl, 1 mm EGTA, protease inhibitors cocktail, 0.5% NP‐40 and 10% glycerol were incubated immunoprecipitated with Protein G Sepharose beads coupled with the relevant antibody at 4 °C overnight 31. Immunoprecipitated proteins were analyzed by immunoblotting. GTP‐bound RHOA was determined by the RHO Activation Assay Kit (Millipore) according to the supplier's protocol.

For immunoblotting, cell lysates and immunoprecipitates were subjected to SDS/PAGE and blotted to PVDF membranes subsequently probed with the following primary antibodies: α‐ACTIN, α‐ROKα, α‐RHOA, α‐BRAF, and α‐pS3‐COFILIN (all Santa Cruz Biotechnology, Santa Cruz, CA, USA); α‐COFILIN (Abcam, Cambridge, UK); α‐RAF1, α‐αCATENIN, α‐βCATENIN, α‐p120CATENIN, and α‐VEC (all BD Pharmingen); and α‐pT202/Y204‐ERK, α‐ERK, α‐pY1173VEGFR2, α‐pY949 VEGFR2, α‐VEGFR2, α‐pS1177eNOS, α‐eNOS, α‐pS473‐AKT, and α‐pT508/T505‐LIMK (all Cell Signaling, Cambridge, UK). After incubation with the appropriate secondary antibody, the antigens were visualized by ECL (Pierce, Thermo Fisher Scientific, Waltham, MA, USA). Immunoblots were quantified using the imagej or the Image Lab (BioRad, Hercules, CA, USA) software.

The GST‐tagged GTPγS‐loaded Rho was generated by expressing pGEX2‐GST‐Rho (1–181) 53 in BL21 *E. coli*. Expression was induced with 0.1 mm IPTG overnight at 18 **°**C and GST‐Rho was harvested from cleared bacterial lysate (50 mm Tris, pH = 7.5, 150 mm KCl, 2 mm MgCl2) by glutathione affinity chromatography (GE Healthcare). GTPγS loading was performed in the elution buffer containing 5 mm EDTA by incubating 35 μm GST‐Rho with 7 μm Rho‐GEF, DBS, and 2 mm GTPγS (RT, 1 h). After overnight dialysis to remove glutathione, GTPγS‐loaded GST‐Rho was immobilized on the GST‐resin and incubated with pMEC lysates (160 μg on 70 μL 50% beads) overnight at 4 **°**C prior to washing, and immunoblotting.

### Vascular permeability assays

Vascular permeability was determined using Evans blue dye (Miles assay 54). Intradermal injections (20 μL) of recombinant VEGF (400 ng) 24, histamine (1 μg) 11, or thrombin (10 U) 55 were performed 10 min after intravenous (i.v.) injection of sterile Evans Blue dye (100 μL, 1% in PBS). After 20 min, the injection sites were excised and incubated in formamide for 5 days, and the extracted dye was determined by spectrophotometric measurement at 620 nm. Values are expressed in fold increase versus the control injection with PBS.

### Tumor allografts

Allografts (10^6^ LLC‐1 or B16F10 cells 56 in 100 μL PBS) were introduced subcutaneously in the flank of 8–10‐week‐old C57/BL6xSv129 F1 BRAF^F/F^ or BRAF^Δ/Δ^ mice 21. Tumor size was measured using a caliper at the indicated times. Tumor volume was calculated by the formula (4/3*(Π*(Length/2)*(Width/2)^2^). Tumor‐bearing mice were sacrificed 14 (LLC1) or 13 (B16F10) days after injection of tumor cells.

### Extravasation assay

B16F10 melanoma cells (1 × 10^6^) stained with CellTracker™ Orange CMRA Dye (Molecular Probes, Invitrogen Life Technologies) were injected in the tail vein of 8‐week‐old mice. After 2 h, two mice of each genotype were sacrificed and analyzed to control for similar lodging in the lung microvasculature. Forty‐eight hours after injection, images of total lungs were acquired with the stereomicroscope Zeiss SteREO Discovery V.12 and the number of cells in the extravasation area of each of the three lobes of the lungs was quantified using the imagej software (NIH) 57. The numbers in the plots represent the mean ± SEM of the indicated biological replicates.

### Transendothelial migration assay

The pMECs were cultured on fibronectin‐coated inserts (8‐μm pore size) for 48 h before B16F10 melanoma cells (2 × 10^5^) stained with CellTracker™ Orange CMRA Dye were added to the upper chamber and incubated for 6 h with FBS (1.25%) plus VEGF (200 ng·mL^−1^), thrombin (10 U·mL^−1^), or histamine (100 μm). Experiments were performed in triplicates and four different areas per well were counted; the integrity of pMECs monolayers was determined by crystal violet staining.

### Statistical analysis

Quantitative data are presented as mean ± SD or mean ± SEM as indicated in the figure legend. Pairwise comparisons were performed by Student's *t*‐test (two‐tailed), respectively.

## Conflict of interest

The authors declare no conflict of interest.

## Author contributions

BC designed and performed experimental work and data interpretation, and wrote the first draft. RW, CD, and KE designed and performed experimental work and data interpretation. AV, TH, BM, KK, SF, and ED performed experimental work and data interpretation. MB designed and conceptualized the study, analyzed and interpreted data, and wrote the manuscript.
